# Testing the Growth Rate Hypothesis in Two Wetland Macrophytes Under Different Water Level and Sediment Type Conditions

**DOI:** 10.3389/fpls.2020.01191

**Published:** 2020-08-05

**Authors:** Cong Hu, Feng Li, Nan Yang, Yong-hong Xie, Xin-sheng Chen, Zheng-miao Deng

**Affiliations:** ^1^ Key Laboratory of Agro-ecological Processes in Subtropical Region, Institute of Subtropical Agriculture, Chinese Academy of Sciences, Changsha, China; ^2^ School of Environment and Life Science, Nanning Normal University, Nanning, China; ^3^ Dongting Lake Station for Wetland Ecosystem Research, Institute of Subtropical Agriculture, Chinese Academy of Sciences, Changsha, China; ^4^ College of Architecture and Urban Planning, Hunan City University, Yiyang, China

**Keywords:** water level, sediment type, growth rate hypothesis, plant stoichiometry, *Carex brevicuspis*, *Polygonum hydropiper*

## Abstract

The growth rate hypothesis (GRH) states that a negative correlation exists between the growth rate and N:P and C:P ratios, because fast-growing organisms need relatively more phosphorus-rich RNA to support their high rates of protein synthesis. However, it is still uncertain whether the GRH is applicable in freshwater wetlands. Several studies have shown that water level and sediment type are key factors influencing plant growth and plant C:N:P characteristics in freshwater wetlands. Thus, this study aimed to elucidate the influence of these factors on plant growth and test the GRH under varying water levels and sediment conditions. We designed a controlled experiment at three water levels and under three sediment types using the two dominant plants (*Carex brevicuspis* and *Polygonum hydropiper*) in the East Dongting Lake wetland, and we further investigated the relative growth rate (RGR); concentrations of total carbon (TC), total nitrogen (TN), and total phosphorus (TP); and plant stoichiometry (ratios of C:N, C:P, and N:P) in the aboveground and belowground parts and whole plants in both species. Results demonstrated that the RGR and TC of both species decreased significantly with decreasing sediment nutrient supply and increasing water level. However, TN and TP of both species were markedly higher at high water levels than at low water levels; furthermore, these were significantly higher on clay than on the other two sediment types at each water level. The C:N and C:P ratios of both species decreased with increasing sediment nutrient supply and water level, whereas N:P decreased in both species with increasing sediment nutrient supply. The aboveground part of *C. brevicuspis* as well as the aboveground part and whole plant of *P. hydropiper* were negatively correlated with N:P, which is consistent with the GRH. However, the relationship between the belowground RGR and N:P of these species was inconsistent with GRH. Therefore, the water level and sediment type and their interaction significantly influenced plant RGR and C:N:P characteristics. The RGR and plant stoichiometry differed significantly between plant organs, indicating that the GRH needs refinement when applied to wetland macrophytes.

## Introduction

The growth rate hypothesis (GRH) proposes that fast-growing organisms have low N:P and C:P ratios due to the relatively high demand for phosphorus-rich RNA to support rapid protein synthesis (Acharya et al., 2004). Various comprehensive reviews confirmed that nutrient-rich plants tend to have low N:P ratios, and supported the validity of GRH in the realm of vascular plants, as N concentration in vascular plants tends to increase less than P concentration (Wright et al., 2005; Kerkhoff and Enquist, 2006; Yu et al., 2012). However, opposite results were also reported (Peng et al., 2010; Loladze and Elser, 2011). For instance, Matzek and Vitousek (2009) found that there was no link between growth rate and leaf N:P for pine species, because RNA comprises only a small proportion of total P (TP) to strongly influence leaf P concentration. To date, the GRH hypothesis has been tested in a variety of ecosystems, and at relatively large scales (Güsewell, 2004; McGroddy et al., 2004; Lovelock et al., 2007); however, it is still uncertain whether it is applicable in freshwater wetlands.

Water level is the dominant factor influencing nutrient cycling and the structure of wetland plant communities (Lowe et al., 2010; Sardans et al., 2012; Saaltink et al., 2018). It can constrain the growth and nutrient availability to wetland macrophytes mainly by limiting oxygen (Casanova and Brock, 2000) and light (Cronin and Lodge, 2003; Miao and Zou, 2012) availabilities and by changing soil nutrient cycling (Steinman et al., 2012; Wang et al., 2015a). For example, *Carex brevicuspis*, which has a relatively low growth rate, was reported to have high N:P ratio and high N and P concentrations at high water levels, both probably caused by anoxic stress (Li et al., 2018a). On the contrary, Li et al. (2013) found that increasing water level decreased the relative growth rate (RGR) of *Potamogeton malaianu* without affecting its N:P ratio and concentrations of N and P. This inconsistency indicates that the relationship between RGR and N:P ratio at different water levels and for different plant species is far from clear. Moreover, high water levels significantly affect soil nutrient availability by changing its geochemical cycle as well as the activity of soil microorganisms (Niedermeier and Robinson, 2007; González Mace et al., 2016), thereby determining plant stoichiometry. For example, the soil mineralization process of organic N results in the accumulation of ammonium under anaerobic conditions, further affecting the N cycle of plants in wetlands (Hefting et al., 2004). Soil P availability also increases due to the reduction of iron, which releases soluble P into the soil (Bridgham et al., 1998; Saaltink et al., 2018). To date, many studies have focused on the effects of water level on plant growth and distribution (Madsen et al., 2001; Li et al., 2012). However, the response of plant stoichiometry to varying water levels is still uncertain (Cao et al., 2011; Yuan et al., 2013). Results from the few studies conducted so far are also inconsistent (Miao and Zou, 2012; Li et al., 2013), indicating that changes in plant stoichiometry in response to water level might be species-specific and needs to be further studied.

Sediment type substantially affects plant growth rate and stoichiometry (Luo et al., 2010; Li et al., 2018a). Plants with high nutrient concentrations are able to extend their roots and enhance root uptake rate, thereby enhancing nutrient absorption abilities (Fransen et al., 2001). For instance, plant RGR and concentrations of N and P in sandy sediments are lower than that in clay sediments due to the limited nutrient availability (Li et al., 2015). However, the nutrient-rich sediment had no significant effect on the relative growth rates of *Elodea canadensis* and *Callitriche cophocarpa* possibly due to their low nutrient requirements (Madsen and Cedergreen, 2002). Indeed, the relationship between sediment type and plant stoichiometry is often affected by water level in wetlands (Xie et al., 2009; Li et al., 2017a). The roots of wetland plants usually display contrasting properties to adjust to infertile or flooded environments, and higher water levels commonly further limit plant nutrient absorption (Xie et al., 2009). Therefore, it is difficult to predict the effects of water level and sediment type on plant stoichiometry based on single factors. Although the changes in plant stoichiometry in different sediment types have been widely studied (Morse et al., 2004; Li et al., 2018a), few studies have focused on their interaction with plant C:N:P stoichiometry.

*Carex brevicuspis* and *Polygonum hydropiper* are dominant species in the vegetated zone of the East Dongting Lake wetland. *C. brevicuspis* is a perennial rhizomatous clonal plant widely distributed at low elevations (23–30 m). The belowground meristems of *C. brevicuspis* can produce long rhizomes (2–25 cm long), which are more capable of obtaining resources under stressful conditions, and short rhizomes (< 1 cm long), which are better at using resources in favorable patches. *P. hydropiper* is an annual herb forming patches embedded in stands of *C. brevicuspis*, generally sensitive to flooding stress and inhabiting elevated sites over shallow flooded habitats. Compared to *P. hydropiper*, *C. brevicuspis* has a wider optimal hydrological niche in the East Dongting Lake wetland (Chen et al., 2014; Li et al., 2018a). In this study, we investigated the interactive effects of water level and sediment type on the growth performance and stoichiometry of *C. brevicuspis* and *P. hydropiper.* These two dominant species were planted under three water levels (-30 cm, 0 cm, and 30 cm relative to the soil surface) and three sediment types (clay, sand, and a mixture of sand and clay at a 1:1 volume ratio) in a factorial design with five replicates. The RGR, total C (TC), total N (TN), TP, and C:N, C:P, and N:P ratios in the aboveground and belowground parts and in the whole plant of both species were measured for exploring the relationship between RGR and plant stoichiometry. As so, the present study aimed to (1) elucidate how differences in water level and sediment type affect plant growth and plant C:N:P characteristics; and (2) test whether the relationship between RGR and plant C:N:P stoichiometry is consistent with GRH under different water level and sediment type conditions.

## Materials and Methods

### Study Site and Plant Materials

Dongting Lake (28°30′–30°20′ N, 111°40′–113°10′ E) is the second-largest freshwater lake and the most typical river-connected lake in China; it is characterized by large seasonal fluctuations of the water level and sediment heterogeneity (Xie et al., 2007a). The wetlands are usually completely flooded from May to October, while being susceptible to drought from November to April. The mean annual temperature is 16.8°C, with hot summers (June–August, 27.3°C) and cold winters (December–February, 5.8°C). The mean annual precipitation is 1,382 mm, with more than 60% of the rain falling from April to August (Li et al., 2017b).

*Carex brevicuspis* (Cyperaceae) is a typical perennial rhizomatous sedge distributed in eastern mainland China. The plant is usually 20–55 cm in height, and it flowers and bears fruit from April to May, before flooding occurs in the Dongting Lake wetland (Chen et al., 2011). *Polygonum hydropiper* (Polygonaceae) is an annual herb 40–70 cm in height. Both species experience periodic flooding that normally occurs between May and October (Chen et al., 2014).

### Sampling

*C. brevicuspis* was collected in Xiaoxihu and *P. hydropiper* was collected in Dingzidi, both in East Dongting Lake, during March 2016. New ramets were dug up and transported to the Dongting Lake Station for Wetland Ecosystem Research, Chinese Academy of Sciences. The new ramets (about 15 cm in height) were placed in plastic basins (55 cm in length, 33 cm in width, 21 cm in height) filled to a depth of 15 cm with soil (4.01 mg g^-1^ soil organic carbon, 0.48 mg g^-1^ soil TN, and 0.57 mg g^-1^ soil TP) that was collected from a *C. brevicuspis* and *P. hydropiper* mixed community in the East Dongting Lake. After one month, similar-sized plants (4–5 leaves, about 25 cm in height) were selected for the experiment.

### Experimental Design

**Figure 1:**
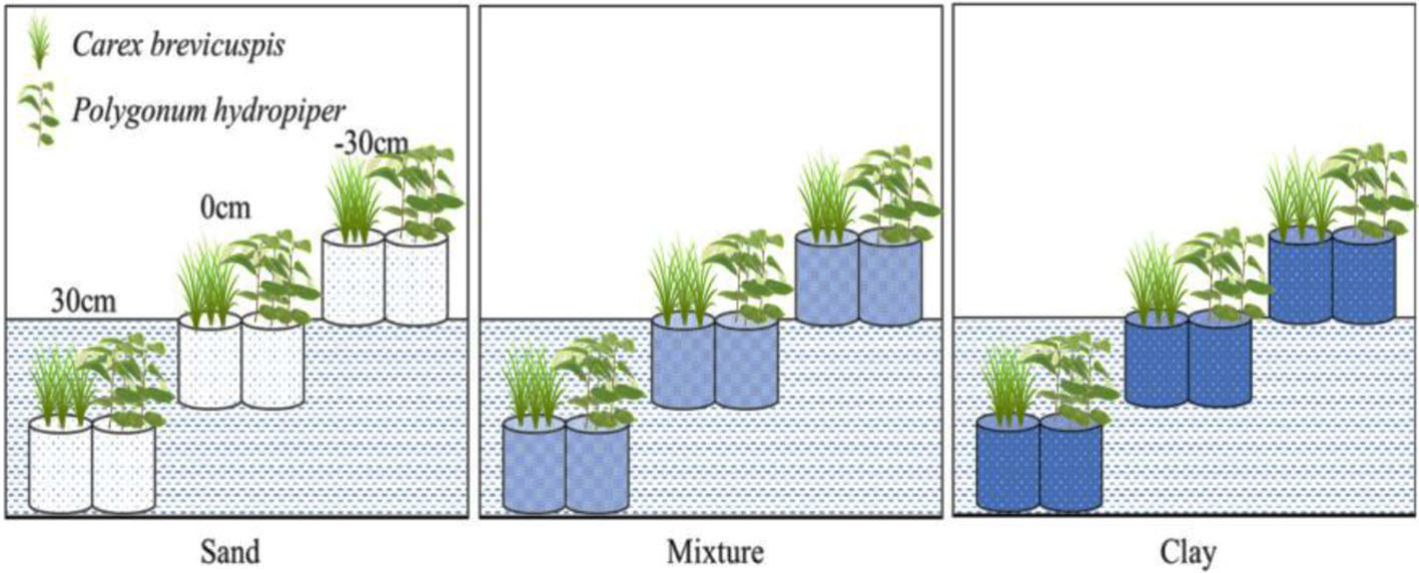
Experimental scheme, showing two plant species (*Carex brevicuspis* and *Polygonum hydropiper*), three sediment types (clay; mixture; sand) and three water levels (-30 cm; 0 cm; 30 cm). Five replicates were made of each treatment.

Before the experiment, ten seedlings of *C. brevicuspis* and ten seedlings of *P. hydropiper* were divided into aboveground and belowground parts, oven-dried, and weighed for the calculation of plant RGR (Li et al., 2016). The experiment combined three water levels (-30 cm, 0 cm, and 30 cm relative to the soil surface) and three sediment types (clay, sand, 1:1 clay–sand mixture) with the two species in a factorial design with five replicates (**Table 1**). Clay was collected from the location described above for ramet germination, and sand was collected from the local river. In the Dongting Lake wetland, most roots of both species are distributed in the top 0–20 cm soil layer (Chen et al., 2014). Therefore, the -30 cm water level was considered the drought treatment, the 0 cm water level was considered the control, and the 30 cm water level was considered the submerged treatment (**Figure 1**). The three sediment types used in the experiment are the main sediment types present in the natural habitat of *C. brevicuspis* and *P. hydropiper* in Dongting Lake. We sampled the clay soil from the same location as plant samples while the sand was collected from the local Xiang River (**Table 1**). On April 2, 2016, the 1,350 similar-sized ramets collected (675 for each species) were transplanted into PVC tubes (30 cm in height and 12 cm in diameter, bottoms enclosed with a nylon netting to prevent soil loss) filled with sediment. Thirty tubes (3 water levels × 2 plant species × 5 tubes) were placed into each of 15 cement pools (1 × 1 × 1 m, five pools per sediment). Three seedlings were planted into each tube for both species, and the experiment started 7 days after planting. Tap water (containing 0.51 μg L^-1^ NH_4_-N, 1.76 μg L^-1^ NO_3_-N, and 0.53 μg L^-1^ PO_4_^3+^-P, pH = 7.2) was completely replaced every two weeks to prevent algal growth (**Figure 1**).

**Table 1:** Soil nutrient concentrations of each sediment type.

Sediment type	SOC (mg g^-1^)	TN (mg g^-1^)	TP (mg g^-1^)
Clay	4.53 ± 0.1^a^	0.52 ± 0.04^b^	0.63 ± 0.03^c^
Mixture	3.81 ± 0.02^a^	0.44 ± 0.01^b^	0.53 ± 0.02^c^
Sand	2.76 ± 0.01^a^	0.26 ± 0.01^b^	0.39 ± 0.01^c^

SOC, soil organic carbon; TN, soil total nitrogen; TP, soil total phosphorus (means ± SE). Different letters indicate significant difference among treatments at 0.05 significance level.

### Harvest and Measurements

All plants were harvested after 4 months of treatment. The roots of each plant were carefully excavated from the PVC tubes, cleaned with tap water, and transported to the laboratory for measurements. Plants in each tube were divided into aboveground and belowground parts, oven-dried at 80°C for 48 h, and weighed.

The RGR (relative growth rate) of the aboveground and belowground parts and of the whole plant were calculated for each species using the following formula:

$$\text{RGR}=\frac{\ln X_{1}-\ln X_{2}}{T},$$

where *X*_1_ and *X*_2_ are the biomass of the aboveground or belowground parts or of the whole plant at the end and start of the experiment, respectively, and *T* is the duration of the experiment (Yuan et al., 2016).

### Total C, N, and P Concentrations

The aboveground and belowground parts and the whole plant of each species in each PVC tube were ground into powder and analyzed for TC and TN using an elemental analyzer (Vario EL III; Elementar, Hanau, Germany). Total P was measured with colorimetric analysis on a TU-1901 spectrophotometer (Beijing Purkinje General Instrument Co., Ltd., Beijing, China) after being pretreated by H_2_SO_4_–H_2_O_2_ digestion (Xie et al., 2007b). Three replicates were used to determine plant C, N, and P concentrations.

### Statistical Analyses

The mean values of the five replicates for each treatment in each pool were used for data analysis. The effect of water level and sediment type on RGR, TC, TN, and TP concentrations and the stoichiometry of the aboveground and belowground parts and whole plant of each species were assessed using a general linear model (GLM). Multiple comparisons of the means were performed using Tukey^’^s test at the 0.05 significance level. All statistical analyses were performed in SPSS 20.0 (SPSS Inc., Chicago, IL, USA).

## Results

### RGRs of *C. brevicuspis* and *P. hydropiper*

**Figure 2:**
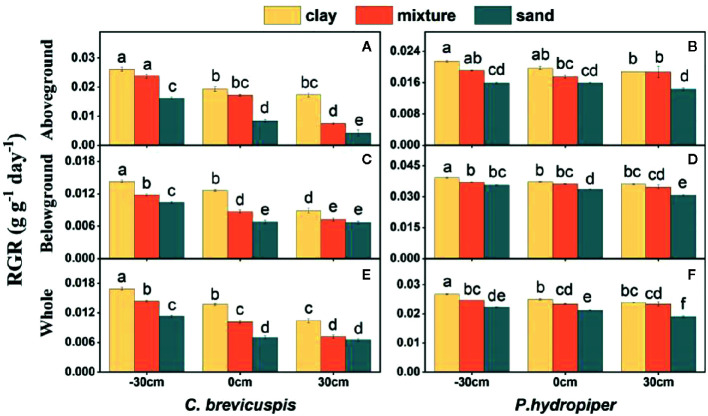
Relative growth rate (RGR) in aboveground part, belowground parts and whole plants of *C. brevicuspis* **(A, C, E)** and *P. hydropiper* **(B, D, F)** in treatments with three sediment types (clay; mixture; sand) and three water levels (-30 cm; 0 cm; 30 cm). Values are means ± SE, with five replications. Different letters indicate significant difference among treatments at 0.05 significance level.

The RGR of the aboveground and belowground parts and whole plants of *C. brevicuspis* and *P. hydropiper* were significantly affected by water level, sediment type, and their interaction (**Table 2**; **Figure 2**). The RGR decreased significantly with increasing water levels in all sediment types, and the highest values of both species were found in the -30 cm water level + clay treatment while the lowest values were found in the 30 cm water level + sand treatment.

**Table 2:** Summary of general linear model (GLM) on plant relative growth rate (RGR), concentrations of TC, TN, and TP, and ratios of C:N, C:P, and N:P in *C. brevicuspis* and *P. hydropiper* growing in three water levels and three sediment types (*F*-values).

			Water level (W)		Sediment type (S)		W*S	
			SS%	F	SS%	*F*	SS%	*F*
RGR	*C. brevicuspis*	AG	51.85	303.38^***^	43.58	255.04^***^	4.57	13.37^***^
(g g^-1^ day^-1^)		BG	53.97	201.96^***^	40.47	151.47^***^	5.56	10.40^***^
		WP	55.80	336.06^***^	41.55	250.21^***^	2.65	7.98^***^
	*P. hydropiper*	AG	9.43	6.10^***^	83.11	53.85^***^	7.47	2.42^ns^
		BG	37.89	64.41^***^	58.42	99.31^***^	3.69	3.14^*^
		WP	22.48	42.77^***^	73.02	138.95^***^	4.51	4.29^**^
TC	*C. brevicuspis*	AG	17	38.91^***^	72.52	469.93^***^	10.48	11.99^***^
(mg g^-1^)		BG	8.68	17.91^***^	87.25	303.29^***^	4.07	4.2^*^
		WP	15.72	62.91^***^	82.45	329.95^***^	1.83	3.66^*^
	*P. hydropiper*	AG	13.2	20.29^***^	82.99	318.84^***^	3.81	2.929^*^
		BG	9.84	26.94^***^	89.13	322.80^***^	1.03	1.42^ns^
		WP	9.45	31.27^***^	89.03	294.65^***^	1.53	2.53^ns^
TN	*C. brevicuspis*	AG	33.38	30.03^***^	60.37	69.55^***^	6.25	2.81^*^
(mg g^-1^)		BG	12.63	24.42^***^	85.13	490.77^***^	2.23	2.16^ns^
		WP	8.12	22.20^***^	88.13	240.97^***^	3.75	5.13^***^
	*P. hydropiper*	AG	28.33	64.76^***^	62.94	115.56^***^	8.73	9.99^***^
		BG	29.02	19.22^***^	58.89	67.47^***^	12.09	4.00^*^
		WP	19.94	22.78^***^	66.46	75.93^***^	13.6	7.77^***^
TP	*C. brevicuspis*	AG	16.11	62.02^***^	71.69	276.68^***^	12.21	23.50^***^
(mg g^-1^)		BG	50.79	270.96^***^	38.23	235.59^***^	10.98	29.30^***^
		WP	33.13	179.81^***^	59.6	323.43^***^	7.27	19.72^***^
	*P. hydropiper*	AG	18.71	59.13^***^	69.91	191.81^***^	11.38	17.98^***^
		BG	30.62	53.13^***^	59.97	342.97^***^	9.41	8.17^***^
		WP	21.56	95.42^***^	68.69	303.98^***^	9.75	21.582^***^
C:N	*C. brevicuspis*	AG	72.92	39.5^***^	15.79	9.48^***^	11.29	3.06^***^
		BG	72.05	37.61^***^	15.57	13.33^**^	12.39	3.23^*^
		WP	73.45	67.94^***^	15.93	14.74^***^	10.62	4.91^**^
	*P. hydropiper*	AG	40.98	63.78^***^	50.75	64.81^***^	8.27	6.43^**^
		BG	49.27	16.10^**^*	41.17	15.33^**^	9.56	1.56^ns^
		WP	17.88	3.93^*^	58.76	12.91^***^	23.36	2.57^ns^
C:P	*C. brevicuspis*	AG	48.53	79.31^***^	36.82	52.69^***^	14.65	11.97^***^
		BG	83.72	181.61^***^	2.6	8.30^**^	13.69	14.85^***^
		WP	86.36	238.15^***^	6.85	18.89^***^	6.79	9.36^***^
	*P. hydropiper*	AG	12.45	22.58^***^	67.04	65.56^***^	20.51	18.59^***^
		BG	53.85	64.14^***^	35.89	130.48^***^	10.26	6.11^**^
		WP	20.19	47.22^***^	64.59	151.05^***^	15.22	17.80^***^
N:P	*C. brevicuspis*	AG	36.92	5.63^**^	16.94	3.65^ns^	46.14	3.52^*^
		BG	76.08	46.44^***^	3.07	2.5^ns^	20.85	6.36^**^
		WP	69.88	25.98^***^	11.29	4.20^*^	18.82	3.5^*^
	*P. hydropiper*	AG	5.43	8.46^***^	65.4	33.89^***^	29.17	22.73^***^
		BG	19.24	0.75^ns^	21.89	1.43^ns^	58.87	1.15^ns^
		WP	12.95	4.74^*^	54.96	20.11^***^	32.09	5.87^**^

SS, sum of squares; AG, Aboveground; BG, Belowground; WP, Whole plant; ns= not significant; *P < 0.05, **P < 0.01, ***P < 0.001.

### Total C, N, and P Concentrations

**Figure 3:**
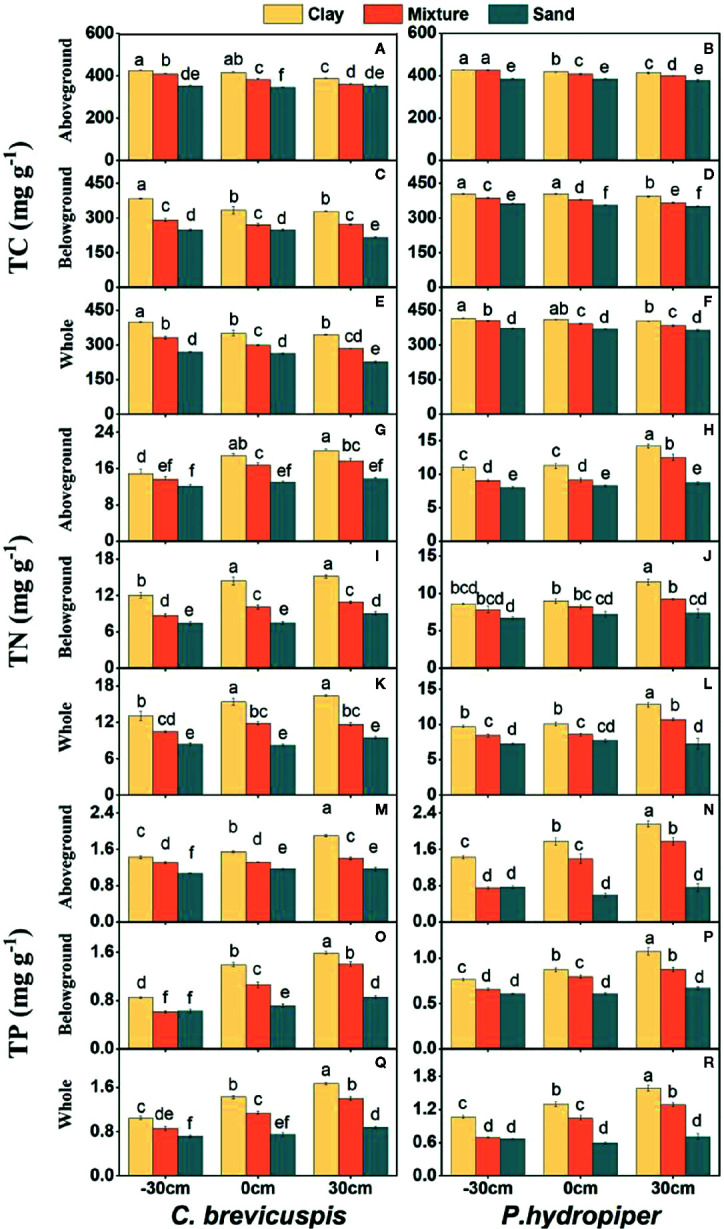
Concentrations of TC **(A–F)**, TN **(G–L)**, and TP **(M–R)** (means ± SE) in aboveground part, belowground parts and whole plants of *C. brevicuspis* and *P. hydropiper* growing in three sediment types (clay; mixture; sand) and three water levels (-30 cm; 0 cm; 30 cm). Different letters indicate significant differences among treatments (*P* < 0.05).

Both water level and sediment type had significant effects on TC, TN, and TP concentrations in the aboveground and belowground parts and whole plants of both species (*P* < 0.001) (**Table 2**). The highest TC concentrations in the aboveground and belowground parts and whole plants of both species were found in the -30 cm water level + clay treatment and they decreased significantly with decreasing sediment nutrient concentration and increasing water level. The TN and TP concentrations in aboveground and belowground parts and whole plants of both species were highest in the 30 cm water level + clay treatment, and they decreased significantly with decreasing sediment nutrient concentration and water level (**Figure 3**).

### C, N, and P Stoichiometry Ratios

**Figure 4:**
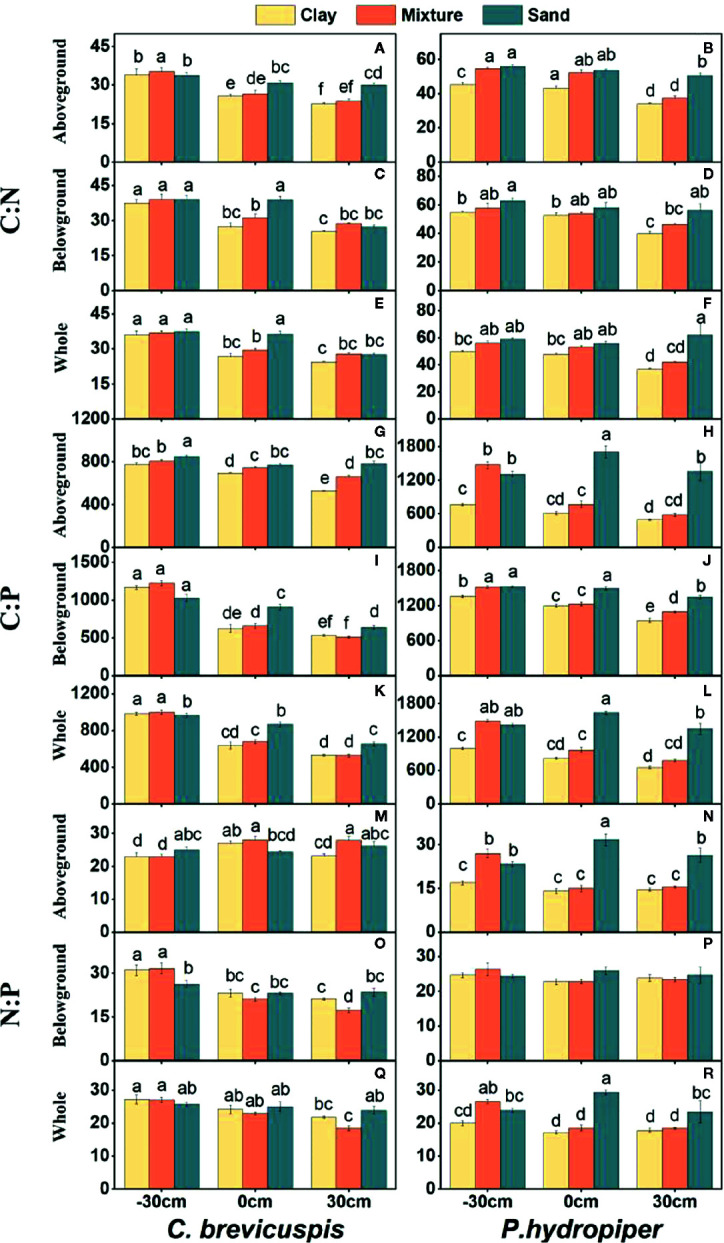
Ratios of C:N **(A–F)**, C:P **(G–L)**, N:P **(M–R)** (means ± SE) in aboveground and belowground parts and the whole plants of *C. brevicuspis* and *P. hydropiper* growing in three sediment types (clay; mixture; sand) and three water levels (-30 cm; 0 cm; 30 cm). Different letters indicate significant differences among treatments (*P* < 0.05).

Water level and sediment type significantly affected C:N and C:P ratios in the aboveground and belowground parts and whole plants of *C. brevicuspis* and *P. hydropiper* (**Table 2**). The C:N and C:P ratios in the aboveground and belowground parts and whole plants of both species decreased with increasing sediment nutrient supply and water level. The highest N:P ratios in the aboveground and belowground parts and whole plants of *P. hydropiper* were found in the 0 cm + sand treatment. The highest N:P ratio in the aboveground part of *P. hydropiper* was found in the 0 cm + mixture treatment and in the belowground part and whole plant were found in the -30 cm + mixture treatment (**Figure 4**).

### Relationships of RGR With C, N, and P Stoichiometry

**Figure 5:**
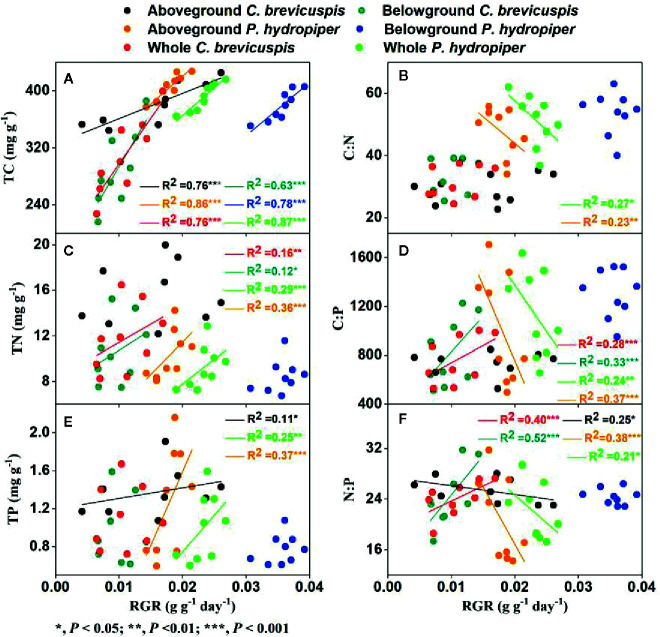
Relationships between relative growth rate (RGR) and concentrations of TC **(A)**, TN **(C)**, TP **(E)**, and ratios of C:N **(B)**, C:P **(D)**, N:P **(F)** (means ± SE) in aboveground and belowground parts and the whole plants of *C. brevicuspis* and *P. hydropiper*.

In *C. brevicuspis*, the RGR of the aboveground part was positively correlated with TC and TP concentrations and negatively correlated with N:P ratio, while the RGR of the belowground part and whole plant were positively correlated with TC and TN concentrations and with C:P and N:P ratios (**Figure 5**).

In *P. hydropiper*, the RGR of the aboveground part and whole plant were positively correlated with the TC, TN, and TP concentrations and negatively correlated with the C:N, C:P, and N:P ratios. The RGR of the belowground part was only positively correlated with TC concentration (**Figure 5**).

## Discussion

The RGR of the aboveground and belowground parts and whole plants of both species decreased significantly with decreasing sediment nutrient concentrations and increasing water levels, indicating that water level, sediment type, and their interaction had a significant effect on plant growth performance (Emery et al., 2001; Xie et al., 2009; Luo et al., 2010). The negative effect of high-water levels on plant growth has been reported in many studies, and it has been mainly attributed to the anaerobic environment and reduced soil redox potential, *Eh* (Sorrell et al., 2000; Steinman et al., 2012). In some of the treatments conducted in the present study, e.g., 0 cm water level + mixture and 30 cm water level + clay, the similar growth performance of the aboveground parts of *C. brevicuspis* indicated that the negative influence of water level on plant growth could be ameliorated in nutrient-rich conditions, as supported by other studies (Wheeler, 1999; Xie et al., 2009). Nutrient availability may increase plant root respiration and root diameter and help plants to acclimate to high water level conditions (Xie et al., 2009; Chen et al., 2016).

The TC concentrations in the aboveground and belowground parts and whole plants of both species decreased significantly with increasing water levels, which was consistent with previous studies (Li et al., 2013; Yuan et al., 2016). High water levels decrease plant photosynthesis, thus leading to a reduction in the synthesis of non-structural carbohydrates in plant tissues (Cao et al., 2009; Su et al., 2016). Plant C balance can be characterized by tissue concentrations of non-structural carbohydrates. When C supply from photosynthesis exceeds the plant’s demand for growth, a large amount of non-structural carbohydrates will accumulate to support future growth. By contrast, when C demand exceeds the C supply, non-structural carbohydrates will only slightly accumulate (Wang et al., 2018). Similar to RGR, plant C concentrations in both species were also higher in the clay treatment than in other sediment types, as soil nutrients are the main determinants of plant nutrient concentrations and therefore influence plant growth (Li et al., 2017b). Wang et al. (2015b) and Zeng et al. (2017) also reported that nutrient-rich sediment conditions result in high C concentration.

The TN and TP concentrations in the aboveground parts of both species were higher compared with those in the belowground parts and whole plants. As described in previous studies (Li et al., 2013; Jing et al., 2017), this phenomenon can be explained by the presence of large amounts of rubisco in the photosynthetic organs (Reich et al., 2004). The TN and TP concentrations in the aboveground and belowground parts and whole plants of both species increased, while C:N and C:P ratios decreased with increasing water level, which was consistent with previous studies (Cronin and Lodge, 2003; Li et al., 2013). For example, TN and TP concentrations of *Cladium jamaicense* increased significantly when water levels increased from 20 to 60 cm (Miao and Zou, 2012). In this study, plants were submerged in 30 cm of water, where light availability was low. The light conditions at the -30 cm water level lead to lower leaf N, probably due to the dilution of available N by increased amounts of fixed C (Cronin and Lodge, 2003). Therefore, lower N and P availability for plant photosynthesis will lead to high plant N and P concentrations. Another study also confirmed that the biomass accumulation of *C. brevicuspis* increased with increasing elevation, while plant TN and TP concentrations decreased, which might have accounted for the dilution effect by which fast-growing plants allocate more N and P to their photosynthetic tissues to support high carbon dioxide assimilation (Yan et al., 2006; Li et al., 2018b). Water level can also influence plant nutrient absorption by changing soil biogeochemical processes (Steinman et al., 2012; Recha et al., 2013). For instance, ammonification is the dominant process at high water levels (Hefting et al., 2004), and it enhances the concentration of available N, promoting plant N absorption (Kaštovská and Šantrůčková, 2011). In addition, soil anoxia can reduce iron plaque formation on roots at high water levels, and thus promote plant P uptake (Saaltink et al., 2018).

At the same water level, the higher TN and TP concentrations and lower C:N, C:P, and N:P ratios in the aboveground and belowground parts and whole plants of both species on the clay sediment indicated that sediment nutrients mainly affect plant nutrients, which could further influence plant stoichiometry (Garbey et al., 2004; Chen et al., 2013; Li et al., 2014). In this study, sediment N and P concentrations in the clay sediment were 2.0 and 1.6 times higher than those in the sand sediment, leading to higher plant N and P concentrations. Moreover, it has been reported that high sediment nutrient levels can promote plant growth and enhance plant nutrient concentrations (Fraser and Feinstein, 2005; Güsewell, 2005). A high clay content would therefore promote soil N mineralization and plant N absorption, while a high sand content allows a higher rate of P leaching (Cross and Schlesinger, 2001).

The N:P ratio in the aboveground parts of both species and whole plant of *P. hydropiper* were negatively correlated with their corresponding RGR, thus supporting the GRH and being consistent with previous studies (Niklas et al., 2005; Niklas, 2006; Ågren, 2008; Cernusak et al., 2010). Ågren (2004) reported that P limited *Betula pendula* seedlings, which displayed decreased N:P at high RGR, supporting the GRH. As a possible explanation, Sterner and Elser (2002) proposed that organisms have to make a relatively large investment in P-rich ribosomes and rRNA to support the rapid protein synthesis associated with fast growth. However, opposite results were found in other studies (Cernusak et al., 2010; Peng et al., 2010). One possible reason for these inconsistent results might be that some plants can store extra nutrients and thus change the relationship between the RGR and the N:P ratio (Jing et al., 2017). Matzek and Vitousek (2009) also showed that plant protein:RNA ratio, but not leaf N:P ratio, was significantly negatively correlated with plant growth rate.

The relationship between RGR and plant stoichiometry in the belowground parts of both species and whole plant of *C. brevicuspis* suggests that the GRH is not valid in these cases, indicating that the applicability of this hypothesis might depend on plant organ and species. In fact, another study reported that the GRH was not consistent with the growth of various organs (Jing et al., 2017). One probable reason might be that a change in environmental factors may lead to the allometric growth of different organs, and the stoichiometry of roots is more sensitive to environmental changes than that of leaves (Minden and Kleyer, 2014; Schreeg et al., 2014). For instance, Jing et al. (2017) confirmed that N addition significantly increased the N:P ratio and RGR of *Pinus tabuliformis* roots in N-limited regions, resulting in a positive relationship between the RGR and N:P ratio of roots. Another reason might be that plants have developed survival strategies other than growth (e.g., storage and defense) that require N and P, in which case a decreasing N:P ratio with increasing growth rate should not necessarily be expected (Matzek and Vitousek, 2009). In addition, plants can store P in vacuoles, allocate N to the production of chemical defenses, or invest different N:P ratios in different organs, all of possibly explaining why P concentration is not greater in fast-growing plants (Méndez and Karlsson, 2005; Peñuelas and Sardans, 2009). However, our results were inconsistent with previous studies (Ågren, 2004; Yu et al., 2012). For instance, Yu et al. (2012) confirmed that the GRH was valid for the roots of three grass plants in the grasslands of Inner Mongolia, and they also proposed that analysis of the relationship between RGR and N:P ratio should consider the N in ribosomes of vascular plants.

In addition, the RGR of the aboveground and belowground parts and whole plant of *C. brevicuspis* were lower than that of *P. hydropiper*, while the N:P ratios in the aboveground and belowground parts and whole plant of *C. brevicuspis* were relatively higher compared with those of *P. hydropiper*. These differences between the two species might be related to the higher tolerance of *C. brevicuspis* to water stress and drought stress compared with *P. hydropiper* (Chen et al., 2014). Namely, stress tolerant plants (characterized by slow growth) have consistently higher N:P ratios than fast-growing plants in wetlands, as the former can focus on the uptake of nitrate while maintaining P reserves due to low internal P demands and efficient conservation (Willby et al., 2001).

This study confirmed that water level, sediment type, and their interaction significantly influence plant growth and plant stoichiometry. Furthermore, we also established that the GRH is valid for the whole plant of *P. hydropiper* and the aboveground parts of both species, but not for whole plant of *C. brevicuspis* and the belowground parts of both species. These results indicate that the GRH needs to be refined for application to macrophytes. However, our study was primarily based on controlled incubation conditions with a relative short duration. Therefore, further studies are still needed to test this hypothesis under long-term natural conditions. In recent years, the area of *C. brevicuspis* and *P. hydropiper* communities in Dongting Lake wetland were seriously reduced due to reduced water levels and anthropogenic disturbances. Therefore, understanding plant growth and stoichiometry characteristics would contribute to the better understanding of macrophytes ecological processes and to establish effective measures for macrophytes’ protection and biodiversity maintenance.

## Data Availability Statement

The original contributions presented in the study are included in the article/supplementary material; further inquiries can be directed to the corresponding authors.

## Author Contributions

CH and FL wrote the manuscript and conducted the technical assays and statistical analyses. NY and Y-HX designed the experiment and edited the manuscript. X-SC and Z-MD contributed to data collection and interpretation. All authors contributed to the article and approved the submitted version.

## Funding

This study was supported by the Joint Fund for Regional Innovation and Development of NSFC (U19A2051), the Youth Innovation Promotion Association of CAS (201861), Key R & D Projects in Hunan Province (2019SK2336) and Changsha Science and Technology Project (kq1907072), the Youth Innovation Development Program of Changsha (kq1802026), and the National Natural Science Foundation of China (31570431).

## Conflict of Interest

The authors declare that the research was conducted in the absence of any commercial or financial relationships that could be construed as a potential conflict of interest.

The reviewer [X-TL] declared a shared affiliation, though no other collaboration, with several of the authors [FL, Y-HX, X-SC, Z-MD] to the handling Editor.
